# Long Noncoding RNA and mRNA m6A Modification Analyses of Periodontal Ligament Stem Cells from the Periodontitis Microenvironment Exposed to Static Mechanical Strain

**DOI:** 10.1155/2022/6243004

**Published:** 2022-11-29

**Authors:** Weifu Sun, Jia Liu, Xu Zhang, Xiaochen Zhang, Jie Gao, Xin Chen, Xian Wang, Wen Qin, Zuolin Jin

**Affiliations:** State Key Laboratory of Military Stomatology and National Clinical Research Center for Oral Diseases and Shanxi Clinical Research Center for Oral Diseases, Department of Orthodontics, School of Stomatology, The Fourth Military Medical University, Xi'an, Shaanxi 710032, China

## Abstract

Periodontal ligament stem cells (PDLSCs) play important roles in orthodontic tooth movement (OTM) and can respond to mechanical stress. Our previous study demonstrated that periodontal ligament stem cells derived from periodontitis tissue (pPDLSCs) are more sensitive to static mechanical strain (SMS) than those derived from healthy tissue (hPDLSCs) and reported the long noncoding RNA (lncRNA) expression profiles of pPDLSCs exposed to SMS. An increasing number of lncRNAs have been reported by various studies to be associated with the osteogenic differentiation of mesenchymal stem cells. Many studies have demonstrated that the n6-methyladenosine (m6A) modification exerts important effects on lncRNA and mRNA regulation of cell behaviors. However, the regulatory effects of lncRNA and mRNA m6A modification on PDLSCs have not been studied. Therefore, we performed an m6A microarray assay with pPLDSCs and hPDLSCs exposed to 12% SMS and found that 143 lncRNAs and 739 mRNAs were differentially methylated. These RNAs were thought to be involved in multiple differentiation and inflammatory responses. Moreover, we found that METTL3, an essential protein in the m6A system, was expressed at lower levels in the strain-exposed pPDLSCs than in strain-exposed hPLDSCs, and METTL3 promoted the osteogenic differentiation of pPDLSCs.

## 1. Introduction

Periodontitis is a complex inflammatory disease characterized by the destruction of periodontal tissue that leads to tooth drift and loss, seriously affecting health and beauty [1]. Patients with periodontitis usually seek orthodontic treatment to achieve better occlusal function and aesthetics [2]. Orthodontic treatment is also associated with minor improvements in periodontal parameters [3]. In addition, orthodontic treatment combined with periodontal tissue regeneration enhances the clinical efficacy of periodontitis treatment [4]. However, periodontal inflammation augments alveolar bone loss and dental root resorption during orthodontic tooth movement [5].

PDLSCs are mesenchymal stem cells with the potential to differentiate into multiple types of cells and show self-renewal capacity [6]. They are considered promising seed cells for periodontium regeneration [7], and they play roles in OTM [8]. However, for patients with periodontitis, PDLSCs affected by an inflammatory microenvironment show lower osteogenic differentiation capacity than PDLSCs derived from healthy periodontium [9]. Thus, the different osteogenic differentiation capacities of hPDLSCs and pPDLSCs may contribute to periodontitis augmentation of alveolar bone loss during OTM.

Mechanical stress is necessary for bone metabolism and OTM [10]. OTM relies on tissue resorption and formation of alveolar bone and periodontal ligaments. Orthodontic force causes osteoclast resorption in areas of compression and osteoblast deposition in areas of tension [11]. However, inappropriate orthodontic force disrupts the balance between osteogenesis and bone resorption, resulting in greater alveolar bone loss [12]. Moreover, our previous study demonstrated that hPDLSCs and pPDLSCs exhibit different sensitivities and adaptations to SMS [9]. Among 6%, 8%, 10%, 12%, and 14% SMS treatments, 12% SMS was the most effective at promoting osteogenic differentiation of hPDLSCs. However, when cells were loaded with 12% SMS, the osteogenic differentiation capacity of the pPDLSCs was significantly lower than that of the hPDLSCs. This result further illustrates the effect of the inflammatory microenvironment and SMS on the biological properties of PDLSCs.

Long noncoding RNAs (lncRNAs), consisting of 200 or more nucleotides, regulate many biological and pathological processes and play roles in periodontitis [13]. Studies have reported amounts of lncRNAs that are differentially expressed between hPDLSCs and pPDLSCs exposed to SMS [14], and some of these lncRNAs affect the osteogenic differentiation capacity of PDLSCs [15, 16]. For example, lncRNA-XIST promotes the osteogenesis of PDLSCs and is expressed at lower levels in strain-exposed pPDLSCs than in strain-exposed hPDLSCs [14]. Additionally, m6A is abundant in mRNAs and lncRNAs. The m6A system consists of “writers” (methyltransferases), “erasers” (demethylases), and “readers” [17]. M6A plays roles in gene regulation and disease processes mediated by methylated RNA [18]. The m6A “writer” METTL3, an essential protein in the m6A system, regulates the multiple differentiation potential of mesenchymal stem cells by targeting JAK1 [19]. Thus, lncRNAs that are methylated by METTL3-induced m6A modification may play roles in the differences between hPDLSC and pPDLSC responses to SMS.

Although many lncRNAs have been reported to be associated with the multiple differentiations of PDLSCs, their mechanisms have been only partially explained. The differences in mRNA and lncRNA m6A modification abundance between pPDLSCs and hPLDSCs and the effects of the m6A modification are still unknown. Therefore, in this study, we aimed to analyze the differences in m6A modification between pPDLSCs and hPDLSCs exposed to SMS and to determine whether METTL3 exerts effects on the osteogenic differentiation of pPDLSCs.

## 2. Materials and Methods

### 2.1. Isolation, Culture, Purification, and Identification of hPDLSC and pPDLSC

hPDLSC samples and pPDLSC samples were obtained from orthodontic patients and chronic periodontitis patients whose premolars and/or third molars needed to be extracted for therapeutic reasons, respectively. All samples were obtained at the Department of Orthodontics and the Department of Periodontology, School of Stomatology, the Fourth Military Medical University. Periodontitis patients were screened according to the following criteria: bleeding on probing; a periodontal pocket <6 mm; 3-4 mm attachment loss; alveolar bone horizontal absorption up to 1/3-1/2 root length as determined on X-ray images. None of the chronic periodontitis patients was treated for acute infection in the previous 6 months, presented with systemic disease, or had a history of smoking, orthognathic surgery, radiotherapy, or chemotherapy. All volunteers signed an informed consent form, and the Fourth Military Medical University Ethics Committee approved this study (Approval Number: 2017(026)). Collagenase type I (Sigma, St. Louis, MO, USA) was used to digest periodontal ligament tissue to obtain free single cells. The tissues and cells were cultured with *α*-MEM (Gibco, Grand Island, NY, USA) supplemented with 10% fetal bovine serum (FBS) (Gibco, Grand Island, NY, USA) and 100 U/mL penicillin and streptomycin (Gibco, Grand Island, NY, USA) at 37°C in 5% CO_2_. The limiting dilution technique was used to purify PDLSCs from other kinds of cells. Single cells were seeded in 96-well plates at a density of 1-cell/well to obtain single-cell-derived colonies. After 7-day culture, the colonies that form colonies were digested, mixed, and seeded in 75 T culture flasks and signed as 1st-generation cells. When reaching 80% confluence, the cells were digested and collected for subculture. All experiments in this study were performed using 2nd- and 4th-generation cells.

Flow cytometric analysis was performed to assess the expression of surface markers of PDLSCs. PDLSCs were washed once with PBS and then digested with 0.25% trypsin-EDTA solution (Hyclone, South Logan, UT, USA). The single-cell suspension was washed twice in 4°C PBS. For the identification of the MSC phenotype, approximately 5 × 10^5^ PDLSCs/200 *μ*L of PBS in each EP tube were incubated with fluorescein isothiocyanate-conjugated monoclonal antibodies for human CD34, CD45, CD106, and stro-1 (BD Biosciences, San Jose, CA, USA) for 1 hour at 4°C in the dark. Then, the cells were washed twice with PBS. Finally, the labeled cells were analyzed by FlowJo, Flow Cytometric Data Analysis Software (TreeStar, Ashland, OR, USA).

Osteogenic differentiation assay was performed to examine the osteogenic differentiation capacity of hPDLSCs and pPDLSCs. PDLSCs were cultured in *α*-MEM with 10% FBS, 100 nM dexamethasone, 50 *μ*g/ml ascorbic acid, and 10 mM *β*-glycerophosphate for 21 days. The medium was changed every 2 days. Calcium accumulation was detected by 2% Alizarin Red (Beyotime, Shanghai, China) staining. After being washed with distilled water three times, stained calcium nodules were identified microscopically.

Adipogenic differentiation assay was performed to examine the adipogenic differentiation capacity of hPDLSCs and pPDLSCs. PDLSCs were cultured in *α*-MEM with 10% FBS, 2 *μ*g/ml insulin, 0.5 mM isobutylmethylxanthine, and 0.5 *μ*M dexamethasone for 21 days. The medium was changed every 2 days. Oil red O (Beyotime, Shanghai, China) staining was performed, and then lipid droplets were identified microscopically.

### 2.2. SMS Loading

Cells were seeded into collagen I-coated 6-well BioFlex plates (Flexcell International, Burlington, NC, USA). When reaching 95% confluence, a Flexcell Tension Plus system (FX-4000 T, Flexcell International) was utilized to load the cells with 12%, 0.1 Hz SMS for 12 h. The pPDLSCs and hPDLSCs were treated the same way.

### 2.3. RNA Extraction

Total RNA was extracted using TRIzol reagent (Invitrogen, Carlsbad, CA, USA) according to the manufacturer's protocol and then quantified with a NanoDrop ND-1000 (Thermo Fisher Scientific, Waltham, MA, USA).

### 2.4. m6A Modification Microarray Assay

Three total RNA samples from both hPDLSC and pPDLSC cultures were collected for the m6A modification microarray assay. The sample preparation and microarray hybridization were performed according to Arraystar's standard protocols. Briefly, total RNA was immunoprecipitated with an anti-N6-methyladenosine (m6A) antibody (Synaptic Systems, Gottingen, Germany). The modified RNAs were eluted from immunoprecipitated magnetic beads and called the “IP” samples. The “IP” RNAs were labeled with Cy5, as cRNAs in reactions using the Arraystar RNA Labelling protocol. The cRNAs were combined and hybridized onto an Arraystar Human mRNA&lncRNA Epitranscriptomic Microarray (8x60K, Arraystar). After washing the slides, the arrays were scanned with an Agilent Scanner G2505C (Agilent Technologies, Santa Clara, CA, USA).

### 2.5. Microarray Analysis

Agilent Feature Extraction software 11.0.1.1 was used to analyze the acquired array images. The raw intensities of IP (immunoprecipitated fraction, Cy5-labelled) were normalized based on the average log2-scaled spike-in RNA intensities. The “m6A quantity” was calculated as the m6A methylation amount based on the IP (Cy5-labelled) normalized intensities. Differentially m6A-methylated RNAs between two comparison groups were identified by filtering based on fold change and statistical significance (*p* value) thresholds. Hierarchical clustering was performed to show the distinguishable m6A-methylation patterns between samples.

### 2.6. Me-RIP Confirmation

The total RNA samples were immunoprecipitated with an anti-N6-methyladenosine (m6A) antibody (Synaptic Systems, Gottingen, Germany). The modified RNAs were eluted from immunoprecipitated magnetic beads and called the “IP” RNAs. “IP” RNAs were reverse transcribed into cDNA using a SuperScript First-Strand Synthesis Kit (Invitrogen, Carlsbad, CA, USA). qPCR was performed using an Applied Biosystems ViiA 7 Real-Time PCR System. The reaction system included incubation for 10 min at 95°C, followed by 40 cycles at 95°C for 10 s, and 60°C for 1 min. The “m6A quantity” was calculated using the 2^-*Δ*Ct^ method, where ΔCt = Ct(pPDLSCs, IP) − Ct(hPDLSCs, IP). The specific primers (TaKaRa, Tokyo, Japan) are presented in Table 1. All experiments were carried out in triplicate.

### 2.7. Real-Time qPCR

RT-qPCR was performed using the SYBR Green PCR Kit (Toyobo, Osaka, Japan) and CFX96 Touch Real-Time PCR Detection System (Hercules, CA, USA). The reaction system included incubation for 1 min at 95°C, followed by 40 cycles at 95°C for 5 s, and 60°C for 50 s. The relative level of mRNAs and lncRNAs was calculated by the 2^-*ΔΔ*Ct^ method and normalized to that of GAPDH. The specific primers (Toyobo, Osaka, Japan) are presented in Table 1. All experiments were carried out in triplicate.

### 2.8. Bioinformatics Analysis of Differentially m6A-Methylated RNAs

A Gene Ontology analysis was performed to determine the association between the differentially methylated mRNAs enriched in particular gene ontological functions and GO terms (http://www.geneontology.org); the annotations are reported according to the GO biological process (BP), cellular component (CC), and molecular function (MF) categories. A Kyoto Encyclopedia of Genes and Genomes (KEGG) (http://www.genome.jp/kegg/) pathway analysis was performed to associate the differentially methylated mRNAs enriched in specific biological pathways.

### 2.9. CeRNA Network Construction

To find potential combinations of microRNAs (miRNAs) and mRNAs for identified lncRNAs, in-house miRNA target prediction software based on TargetScan & miRanda was used [20]. By merging the commonly targeted miRNAs, we constructed a competing endogenous RNA (ceRNA) network [21]. The ceRNA network was plotted with Cytoscape software.

### 2.10. Lentivirus Plasmid Transfection

The lentivirus for METTL3 overexpression was recombinant LV5(EF-1aF/GFP&Puro) vector designed and constructed by GenePharma (Shanghai, China). The lentivirus for METTL3 interference was recombinant LV3 (H1/GFP&Puro) vector purchased from GenePharma (Shanghai, China). Cells were transfected with lentiviruses for 24 h and then cultured with the common medium.

### 2.11. Western Blot Analysis

Cells were lysed with RIPA lysis buffer (Beyotime, Shanghai, China) on ice, and the lysates were added to the loading buffer (Beyotime, Shanghai, China) and heated at 100°C for 10 min. Proteins in the lysates were separated by electrophoresis (Bio-Rad, Hercules, CA, USA) and transferred to polyvinylidene fluoride membranes (Millipore, Billerica, MA, USA). The membranes were blocked with a blocking buffer (Beyotime, Shanghai, China) at 25°C for 2 h. Then, the membranes were incubated with primary antibodies (Cell Signaling Technology, Danvers, MA, USA) for 12 h at 4°C and incubated with secondary antibodies (Invitrogen, Carlsbad, CA, USA) for 1.5 h at 25°C. Finally, a high-sensitivity ECL chemiluminescence kit (Beyotime, Shanghai, China) was used to visualize the blots on the polyvinylidene fluoride membranes.

### 2.12. Alizarin Red Staining Analysis

The method of Alizarin Red staining was described in 2.1. The Alizarin Red staining was released by cetyl pyridinium chloride (CPC) (Sigma, St. Louis, MO, USA) and quantified by spectrophotometry at 562 nm.

### 2.13. Statistical Analysis

SPSS 16.0 software (SPSS, San Rafael, CA, USA) was used to analyze the data. All experiments were performed in triplicate, and all data are presented as the means ± standard deviation (S.D.). Student's *t*-test determined statistical significance. A *p* value <0.05 was considered to be statistically significant.

## 3. Results

### 3.1. Identification of hPDLSCs and pPDLSCs

**Figure 1:**
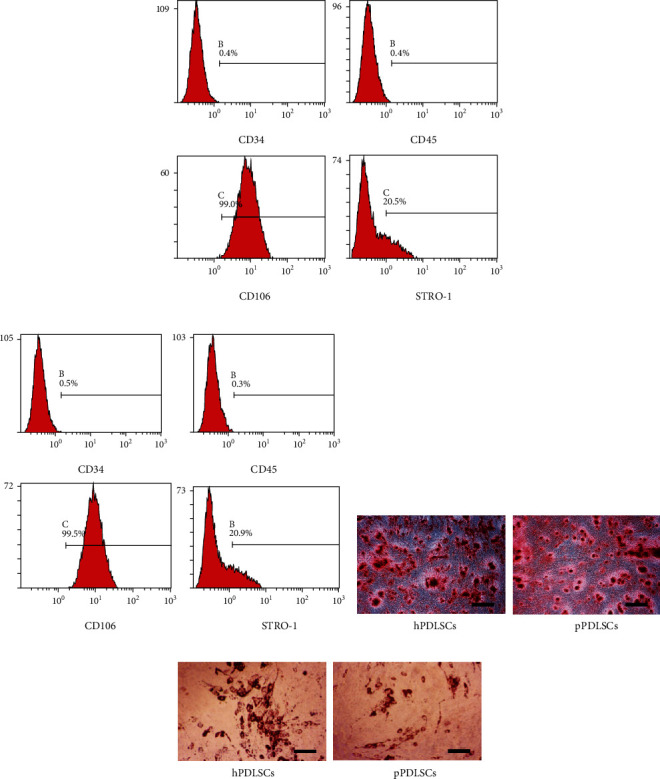
Identification of hPDLSCs and pPDLSCs. The surface markers of hPDLSCs (a) and pPDLSCs (b). Alizarin Red staining (c) and Oil red O staining (d) of hPDLSCs and pPDLSCs.

**Figure 2:**
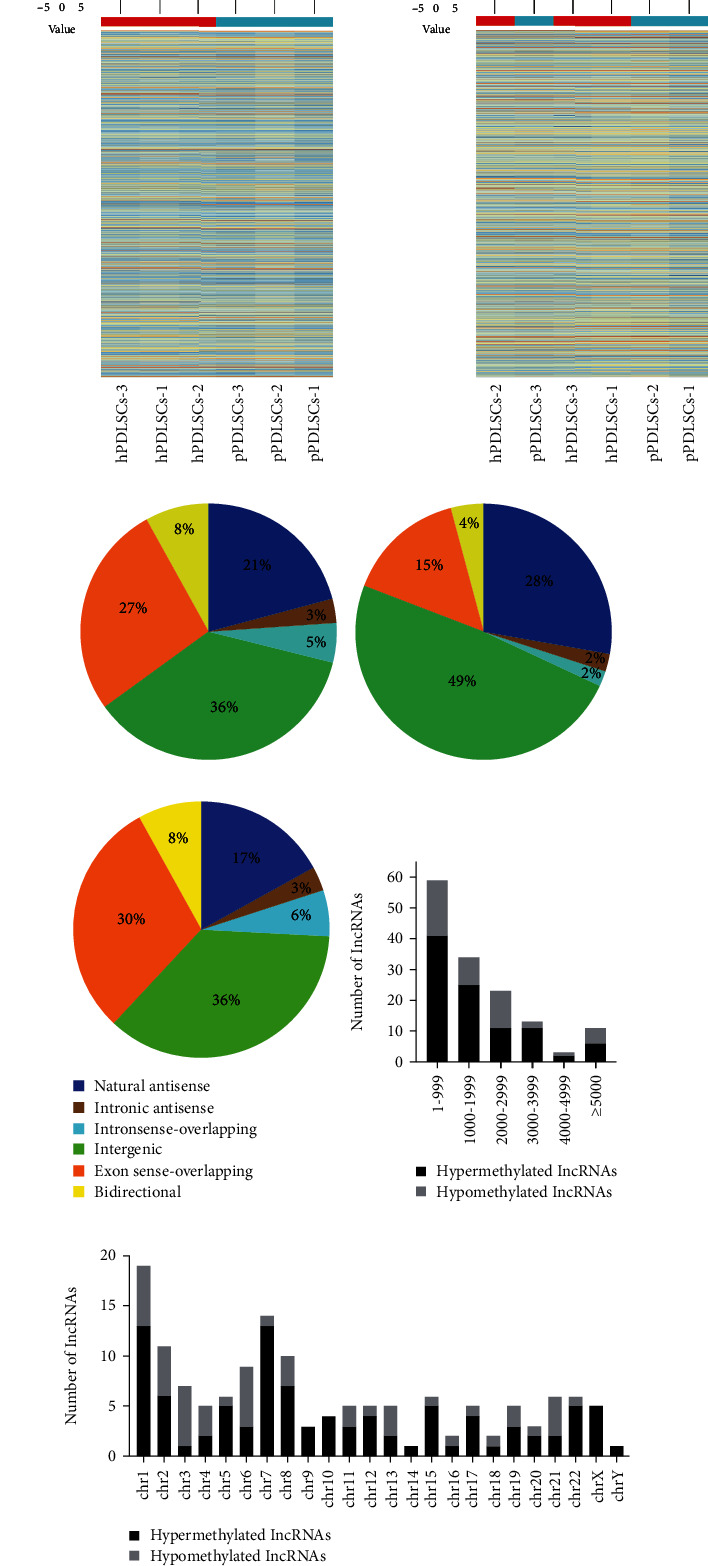
Overview of differentially m6A-methylated lncRNAs and mRNAs in pPDLSCs with SMS. Hierarchical clustering results show differentially m6A-methylated lncRNAs (a) and mRNAs (b). Cool colors represent low levels. Warm colors represent high levels. Positional relationship categories of the differentially methylated lncRNAs (c), hypermethylated lncRNAs (d), and hypomethylated lncRNAs (e). Length of differentially methylated lncRNAs (f). Distribution of differentially expressed M6A lncRNAs on chromosomes (g).

Both hPDLSCs (Figure 1(a)) and pPDLSCs (Figure 2(b)) were positive for CD106 and STRO-1; and negative for CD34 and CD45. And both of them possessed the ability of osteogenic differentiation (Figure 1(c)) and adipogenic differentiation (Figure 1(d)).

### 3.2. Analysis of Differentially m6A-Modified lncRNAs and mRNAs in pPDLSCs under SMS

To investigate the differentially m6A-modified lncRNAs and mRNAs between pPDLSCs and hPDLSCs with SMS, an m6A microarray assay was performed. The distribution of the m6A modification of lncRNAs and mRNAs is shown in a heat map (Figures 2(a) and 2(b)). We found 96 hypermethylated lncRNAs and 535 hypermethylated mRNAs (*p* < 0.05, fold change>2) and 47 hypomethylated lncRNAs and 204 hypomethylated mRNAs (*p* < 0.05, fold change<0.5). The 20 most differentially methylated lncRNAs and mRNAs are shown in Tables 2 and 3.

Based on the positional relationship, the differentially methylated lncRNAs were divided into six categories: natural antisense, intronic antisense, intron sense-overlapping, intergenic, exon sense-overlapping, and bidirectional. The majority of the lncRNAs contained intergenic m6A sites, accounting for 49% and 36% of the hypermethylated lncRNAs and hypomethylated lncRNAs, respectively (Figures 2(c), 2(d), and 2(e)).

We further classified differentially methylated lncRNAs by their length. The lncRNAs that were less than 1000 nt in length accounted for more than one-half of the total lncRNAs (Figure 2(f)). In addition, hypermethylated lncRNAs were distributed on all chromosomes, while hypomethylated lncRNAs were distributed on chromosomes except chr9, chr10, chr14, and chr23 (Figure 2(g)).

### 3.3. Confirmation of Differentially Methylated RNAs

**Figure 3:**
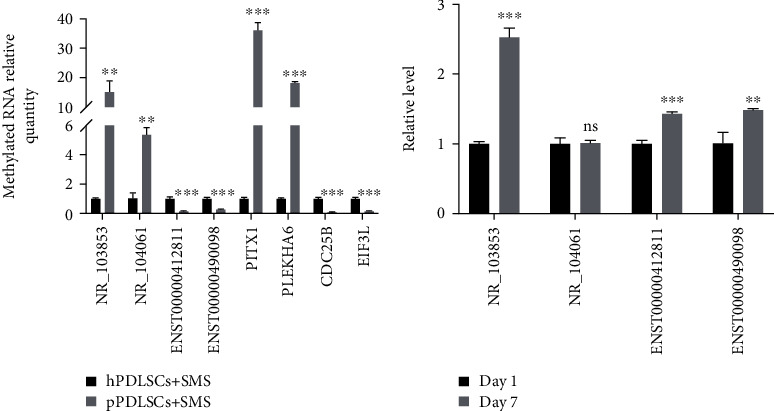
Confirmation of differentially methylated lncRNAs. Two hypermethylated lncRNAs, two hypomethylated lncRNAs, two hypermethylated mRNAs, and two hypomethylated mRNAs were confirmed by RIP-qPCR (a). The relative level of differentially methylated lncRNAs before and after osteogenic induction (b). ^∗^*p* < 0.05, ^∗∗^*p* < 0.01, ^∗∗∗^*p* < 0.001.

To verify the microarray results, Me-RIP and qPCR were performed to evaluate the methylation level of the lncRNAs and mRNAs. We selected two hypermethylated lncRNAs (NR_103853 and NR_104061), two hypomethylated lncRNAs (ENST412811 and ENST490098), two hypermethylated mRNAs (PITX1 and PLEKHA6), and two hypomethylated mRNAs (CDC25B and EIF3L) from among the 20 most differentially methylated RNAs, as shown in Table 2. The quantities of m6A modified NR_103853, NR_104061, PITX1 and PLEKHA6 in the pPDLSCs exposed to SMS were more than those in the hPDLSCs exposed to SMS, and the quantities of m6A modified ENST412811, ENST490098, CDC25B, and EIF3L in pPDLSCs exposed to SMS were smaller (Figure 3(a)). These findings were consistent with the microarray results.

Besides, to verify whether the four lncRNAs were related to osteogenesis, we detected the relative level of these lncRNAs after a 7-day osteogenic induction in pPDLSCs. The results showed that the increased relative level of NR_103853, ENST412811, and ENST490098 suggested a positive correlation between the three lncRNAs and osteogenic differentiation (Figure 3(b)).

### 3.4. Study of Differentially Methylated mRNAs

**Figure 4:**
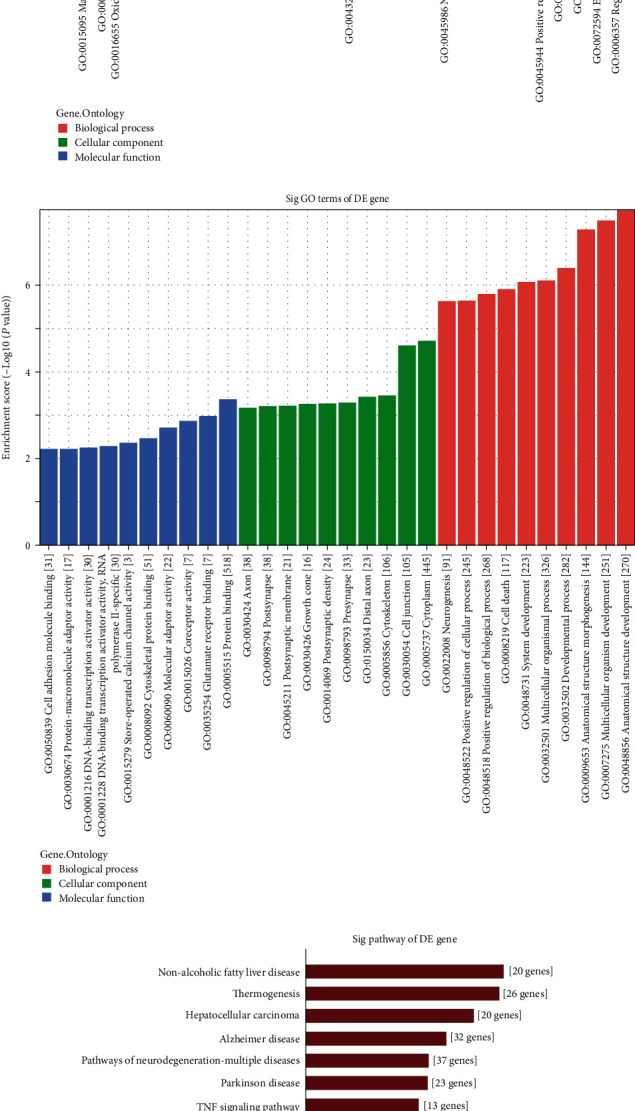
Functional analysis of differentially methylated mRNAs. GO enrichment analysis of hypermethylated mRNAs (a) and hypomethylated mRNAs (b). Pathway analysis of hypermethylated mRNAs (c) and hypomethylated mRNAs (d).

GO and KEGG analyses of these mRNAs were performed to further explore the differentially methylated mRNAs' functions. GO analysis revealed that hypermethylated mRNAs were mainly enriched in beta-tubulin binding, cellular metabolic processes, oxidoreductase activity, etc. (Figure 4(a)). Hypomethylated mRNAs were mainly enriched in the developmental process, cytoskeletal protein binding, store−operated calcium channel activity, etc. (Figure 4(b)).

The KEGG analysis revealed that hypermethylated mRNAs were involved in the TNF signaling pathway, oxidative phosphorylation, etc. (Figure 4(c)). Hypomethylated mRNAs were mainly involved in inflammatory mediator regulation of TRP channels, calcium signaling pathway, etc. (Figure 4(d)).

### 3.5. Construction of a ceRNA Network and Study of Differentially Methylated lncRNAs

**Figure 5:**
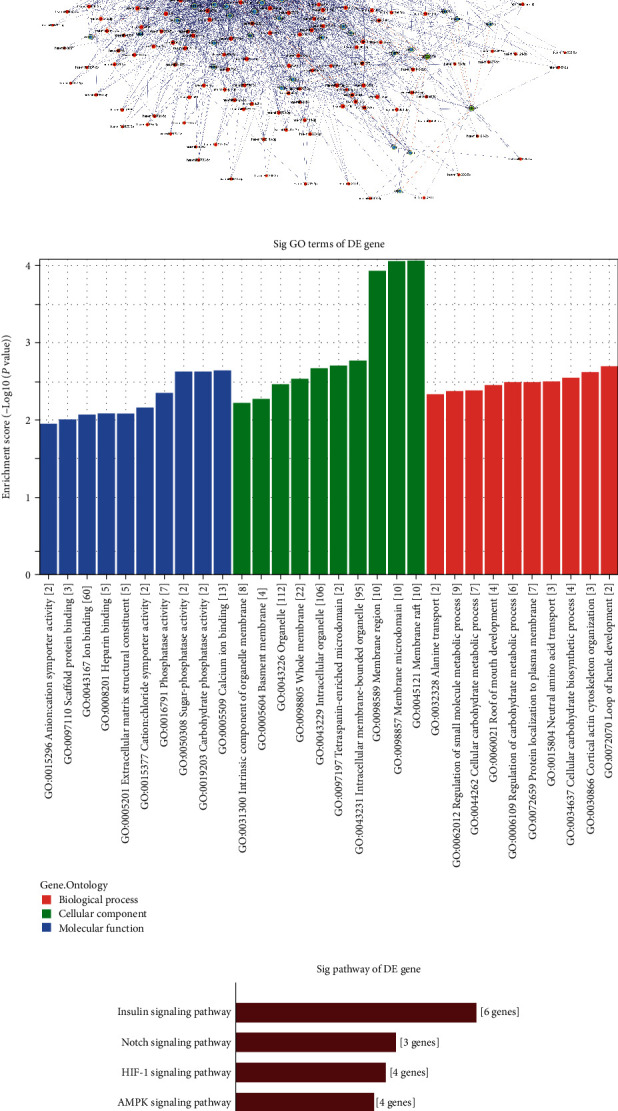
Functional analysis of differentially methylated lncRNAs. CeRNA network with 8 significantly differentially methylated lncRNAs (a). All RNAs are presented by gene symbol. Light green represents hypermethylated lncRNAs. Yellow represents hypomethylated lncRNAs. Light blue represents mRNAs. Red represents miRNAs. GO enrichment analysis (b) and pathway analysis (c) of mRNA-related lncRNAs in the ceRNA network.

Previous studies have reported that lncRNAs can affect cell physiological and pathological processes by many mechanisms, such as miRNA sponging, which regulates mRNA expression. Therefore, we chose the top 5 hypermethylated and top 5 hypomethylated lncRNAs except for lncRNAs which could not act as miRNA sponge among the 20 most differentially methylated lncRNAs to construct a lncRNA-miRNA–mRNA ceRNA network. Among them, ENST00000515377 and ENST00000412811 were not included in the ceRNA network because only the miRNAs that interact with them were predicted but no mRNA was predicted. As a result, the network finally included 8 lncRNAs, 440 miRNAs, and 158 mRNAs (Figure 5(a)). To further evaluate the effects of the chosen 8 lncRNAs on cells via miRNA–mRNA interaction, GO and KEGG analyses of the 158 mRNAs were performed. The GO analysis revealed that these mRNAs were enriched in scaffold protein binding, extracellular matrix structural constituent, phosphatase activity, calcium ion binding, etc. (Figure 5(b)). The KEGG analysis revealed that these mRNAs were involved in phagosomes, the AMPK signaling pathway, the Notch signaling pathway, etc. (Figure 5(c)).

### 3.6. METTL3 Is Differentially Expressed in Stained pPDLSCs and Promotes the Osteogenic Differentiation of pPDLSCs

**Figure 6:**
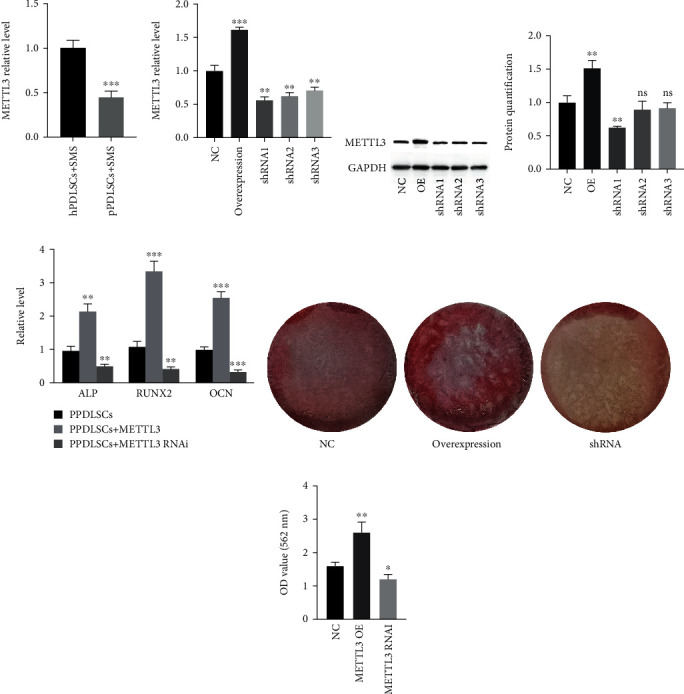
METTL3 is differentially expressed in stained pPDLSCs and promotes the osteogenic differentiation of pPDLSCs. The relative expression level of METTL3 in hPDLSCs and pPDLSCs under strain (a). The effects of lentiviruses on METTL3 were determined by qPCR (b), western blotting (c) and quantification analysis (d). The expression levels of osteogenesis-related genes (ALP, RUNX2, and OCN) after 7 days of osteogenic induction (e). Alizarin Red staining and quantification analysis after 21 days of osteogenic induction (f and g). OE, overexpression. ^∗^*p* < 0.05, ^∗∗^*p* < 0.01, ^∗∗∗^*p* < 0.001 ns *p* > 0.05.

A “writer”, METTL3 is a critical protein in m6A modification, and it has been reported to play roles in mesenchymal stem cell differentiation. To evaluate the difference in METTL3 expression levels between strained hPDLSCs and pPDLSCs, qPCR was performed to determine the METTL3 expression level. The results revealed that METTL3 expression levels were lower in the pPLDSCs exposed to SMS than in hPDLSCs exposed to SMS (Figure 6(a)). To further evaluate the role played by METTL3 in the pPDLSC osteogenic differentiation, we upregulated and downregulated METTL3 expression through lentivirus transfection of pPDLSCs. To prevent off-target short hairpin RNA (shRNA) effects, three different shRNAs against METTL3 were designed. The results revealed that lentiviruses successfully regulated METTL3 at both the mRNA and protein levels. In addition, the most efficient shRNA (shRNA-1) was selected to downregulate METTL3 expression (Figures 6(b), 6(c), and 6(d)). Osteogenic gene (RUNX2, ALP, and OCN) expression levels were measured by qPCR after 7 days of osteogenic induction, and Alizarin Red staining was performed after 21 days of osteogenic induction. METTL3 overexpression increased the osteogenic gene expression level and mineralized bone matrix formation of pPDLSCs. In contrast, the downregulation of METTL3 expression decreased the osteogenic gene expression level and mineralized bone matrix formation of pPDLSCs (Figures 6(e), 6(f), and 6(g)). Taken together, SMS suppressed the osteogenic differentiation of pPDLSCs by downregulating METTL3 expression. The low METTL3 expression level induced by SMS suppressed the osteogenic differentiation of pPDLSCs.

## 4. Discussion

To further determine the mechanism of m6A-mediated mRNAs and lncRNAs in the processes of multiple differentiations of PDLSCs loaded with SMS, we performed an m6A microarray assay of hPLDSCs and pPDLSCs under strain. 96 hypermethylated lncRNAs and 535 hypermethylated mRNAs and 47 hypomethylated lncRNAs and 204 hypomethylated mRNAs were identified. GO and KEGG analyses suggested that these differentially methylated RNAs were associated with various biological and pathological processes. In addition, METTL3, a vital component of the m6A system, has been reported to play roles in multiple differentiations of mesenchymal stem cells. In this study, METTL3 was differentially expressed in hPDLSC and pPDLSCs under SMS, and METTL3 promoted the osteogenic differentiation of pPDLSCs.

The M6A modification is abundant in RNA and is involved in almost every aspect of mRNA metabolism, including processing, export from the nucleus to the cytoplasm, translation, and decay [22, 23]. M6A modification is also involved in the biogenesis and stability of lncRNAs [24]. An increasing number of studies have reported that m6A modification plays roles in mesenchymal stem cell differentiation by methylated lncRNAs and mRNAs [25–27]. M6A-related lncRNAs have also been identified as markers to predict the development and prognosis of cancers [28, 29]. Thus, analyzing differentially methylated mRNAs and lncRNAs may provide references for identifying the mechanism underlying the biological differences between hPDLSCs and pPDLSCs under strain.

To verify the accuracy of the microarray results, we performed meRIP-qPCR to detect the m6A abundance on four selected lncRNAs. The results were consistent with those of the microarray assay. Notably, two modes are usually used to describe the degree of m6A modification of RNA. One is “m6A quantity”, representing the degree of m6A methylation on each RNA, and the other is “m6A level”, representing the percentage of methylated transcripts among all total transcripts. In this study, we screened differentially methylated RNAs based on the m6A quantity on each RNA because previous studies have demonstrated that methylated transcripts play critical roles in biological and pathological processes, in contrast to unmethylated transcripts [30, 31]. In addition, the conditions of the experimental group probably affected the expression level of RNA [14] and thus affected the m6A level. Therefore, this study found that 143 lncRNAs and 739 mRNAs were differentially methylated based on m6A quantity. Bioinformatics analysis revealed that these RNAs are enriched in beta-tubulin binding, cellular metabolic process, developmental process, cytoskeletal protein binding, scaffold protein binding, etc., which have been reported to be associated with the stress response and the development and metabolism of cells [32, 33]. The KEGG analysis also revealed that differentially methylated RNAs were involved in pathways related to the inflammatory response and osteogenic differentiation, including the AMPK signaling pathway and Notch signaling pathway [34–36]. According to these results, differentially methylated RNAs were highly likely to be related to the osteogenic differentiation and different responses of hPDLSCs and pPDLSCs to SMS.

M6A modification plays a crucial role in cell homeostasis, but abnormal m6A modification levels can affect cellular functions and even cause disease [37]. As a “writer” of the m6A system, METTL3 has been reported to be related to stem cell function in increasing studies. Mettl3 inhibits bone marrow stem cell (BMSC) adipogenic differentiation by targeting JAK1 and regulates dental pulp stem cell differentiation by affecting glycolysis [19, 38]. In this study, we found that the total m6A quantity and METTL3 expression level of pPDLSCs exposed to SMS were decreased compared to those of hPDLSCs exposed to SMS, which might be associated with the differences in the osteogenic differentiation capacity between these two types of cells under strain. To further determine the effects of METTL3 on pPDLSCs, METTL3 was overexpressed or downregulated by lentivirus transfection. The results revealed that overexpression of METTL3 promoted osteogenic differentiation of pPDLSCs, while downregulation of METTL3 expression inhibited osteogenic differentiation. This result was similar to the function of METTL3 in bone marrow mesenchymal stem cells [39].

In summary, we identified differentially methylated mRNAs and lncRNAs with an m6A microarray and predicted their functions through GO and KEGG analyses. Bioinformatics analysis demonstrated that the screened RNAs were probably related to the pPDLSC response to SMS. However, the mechanism by which m6A modification regulates cell behaviors by modifying mRNAs or lncRNAs remains to be further studied. In addition, we demonstrated that METTL3 was differentially expressed in hPDSLCs and pPDLSCs treated with SMS and promoted osteogenic differentiation of pPDLSCs. The results of this study supplement the evidence showing that lncRNA and mRNA m6A modification regulates the osteogenic differentiation of PDLSCs and reveal the effects of METTL3 on pPDLSCs, thereby providing a reference for orthodontic treatment of periodontitis patients, to a certain extent.

## 5. Conclusion

In this study, we analyzed differentially m6A-modified lncRNA and mRNA and revealed 96 hypermethylated lncRNAs, 535 hypermethylated mRNAs, 47 hypomethylated lncRNAs, and 204 hypomethylated mRNAs. According to bioinformatics analysis, these lncRNAs and mRNAs were suggested to be closely related to the functional difference between hPDLSCs and pPDLSCs under 12% SMS. Some differentially methylated lncRNAs have been proven to be associated with the osteogenic differentiation of pPDLSCs. In addition, METTL3 was expressed at lower levels in pPLDSCs than in hPDLSCs treated with SMS, and METTL3 promoted the osteogenic differentiation of the pPLDSCs. These results suggested that m6a modification of RNA plays an important role in the osteogenic differentiation of PDLSCs. Further research should consider how METTL3 affects osteogenic differentiation by regulating m6A-modified lncRNAs and mRNAs.

## Figures and Tables

**Table 1:** Primers for qPCR.

Gene symbol	Full name	Sense primer	NCBI ID
NR_103853	EDNRB antisense RNA 1	F: 5′ GAAGGCTTTGTGGAGACCCA 3′	100505518
		R: 5′ GCTTCAAGCACTAGCTTGCC 3′	

NR_104061	Long intergenic nonprotein coding RNA 993	F: 5′ GGCTCTGCTTTGACCTGAAGT 3′	101929520
		R: 5′ TGGTTTCCAGCTCTTTGCAC 3′	

ENST00000412811	—	F: 5′ GGTGGTGGATAAATCGTGGAC 3′	105377043
		R: 5′ CAGCCTGACTGATATGGTGAAAC 3′	

ENST00000490098	—	F: 5′ CATTTCCTTCTGTCTCGCCTC 3′	—
		R: 5′ CCCCGTATGAACTTCCTCCTA 3′	

PITX1	Paired like homeodomain 1	F: 5′CTGTCGTCGCAGTCCATGTTCTC 3′	5307
		R: 5′CGGTGAGGTTGTTGATGTTGTTGAG 3′	

PLEKHA6	Pleckstrin homology domain containing A6	F: 5′ ATTGGCAAGTCAAGCAGGAAGGAG 3′	22874
		R: 5′ GGATGAGGTGGGAGGAGCAGAG 3′	

CDC25B	Cell division cycle 25B	F: 5′ ATGAGATCGAGAACCTCCTGGACAG 3′	994
		R: 5′ GGTCTTGGTGCTTTCCGTCTACTG 3′	

EIF3L	Eukaryotic translation initiation factor 3 subunit L	F: 5′GATCAGCCATCAGCCTGTCAACTC 3′	51386
		R: 5′TGCCACAACCCACAAATTCTCTCC 3′	

METTL3	Methyltransferase 3	F: 5′ TGGGGGTATGAACGGGTAGA 3′	56339
		R: 5′ CCTTTGACACCAACCAAGCAG 3′	

ALP	Alkaline phosphatase	F: 5′ GTGAACCGCAACTGGTACTC 3′	250
		R: 5′ GAGCTGCGTAGCGATGTCC 3′	

RUNX2	RUNX family transcription factor 2	F: 5′ CCCGTGGCCTTCAAGGT 3′	860
		R: 5′ CGTTACCCGCCATGACAGTA 3′	

OCN	Bone gamma-carboxyglutamate protein	F: 5′ CACTCCTCGCCCTATTGGC 3′	632
		R: 5′ CCCTCCTGCTTGGACACAAAG 3′	

GAPDH	Glyceraldehyde-3-phosphate dehydrogenase	F:5′ AGATCCCTCCAAAATCAAGTGG 3′	2597
		R:5′ GGCAGAGATGATGACCCTTTT 3′	

**Table 2:** The 20 most differentially methylated lncRNAs in pPDLSCs under SMS.

Transcript ID	*p* value	Fold change	Regulation	Gene symbol
NR_103853	0.0017254	32.3342345	Up	RP11-318G21.3
NR_149132	0.0254617	12.4902420	Up	MICB-DT
ENST00000515377	0.0001892	12.0728680	Up	CTD-2201E9.2
NR_104061	0.0290754	8.2203114	Up	LINC00993
ENST00000625474	0.0196781	6.5809892	Up	TGFB2-OT1
NR_136740	0.0055997	5.9404909	Up	PAK3
ENST00000528139	0.0056367	5.7134455	Up	RP11-1134I14.8
NR_136745	0.0138980	5.5932792	Up	PAK3
ENST00000481838	0.0297185	4.5138215	Up	MX2
NR_136744	0.0150144	4.3448292	Up	PAK3
ENST00000412811	0.0383625	0.0717941	Down	RP11-761 N21.1
ENST00000630728	0.0000674	0.0733361	Down	TARID
ENST00000445745	0.0056917	0.1067849	Down	PAX8-AS1
ENST00000490098	0.0476888	0.1169665	Down	NTPCR
ENST00000445551	0.0208389	0.1621001	Down	FOXD2-AS1
ENST00000412193	0.0075395	0.2118580	Down	AC093642.4
NR_109991	0.0332787	0.2544106	Down	B3GAT3
ENST00000422956	0.0213980	0.2747888	Down	PAX8-AS1
ENST00000519714	0.0376811	0.2949708	Down	RP11-756 K15.2
NR_137425	0.0130012	0.2952981	Down	RP11-436I9.2

**Table 3:** The 20 most differentially methylated mRNAs in pPDLSCs under SMS.

Transcript ID	*p* value	Fold change	Regulation	Gene symbol
ENST00000265340	0.0000491	113.2070664	Up	PITX1
ENST00000626030	0.0011194	110.9674639	Up	EDNRB
ENST00000272203	0.0005932	24.1329593	Up	PLEKHA6
ENST00000382120	0.0073542	22.7181047	Up	SOD3
ENST00000419673	0.0011690	17.9010111	Up	ARHGAP28
ENST00000279441	0.0083045	16.5808658	Up	MMP10
ENST00000265441	0.0079546	16.5358689	Up	WNT2
ENST00000609571	0.0030226	15.4852525	Up	ATRNL1
ENST00000274063	0.0019242	15.3533649	Up	SFRP2
ENST00000391781	0.0144628	14.5770744	Up	ZNF468
ENST00000361360	0.0002383	0.0086917	Down	POU3F3
ENST00000245960	0.0338174	0.0612121	Down	CDC25B
ENST00000624234	0.0255561	0.0658737	Down	EIF3L
ENST00000397614	0.0345079	0.0705057	Down	RNH1
ENST00000276594	0.0337500	0.0874991	Down	PRDM14
ENST00000467467	0.0256405	0.0985269	Down	WWTR1
ENST00000272521	0.0236208	0.1031436	Down	TMEM177
ENST00000359478	0.0378033	0.1065764	Down	MFAP5
ENST00000355422	0.0355430	0.1080789	Down	GFRA1
NM_001350253	0.0374981	0.1183285	Down	TRMT5

## Data Availability

The data used to support the findings of this study are available from the corresponding author upon request.
